# *De novo* podocytopathy following moderna COVID-19 vaccine: A case report and racial disproportionality in adverse effect reports

**DOI:** 10.3389/fmed.2022.844004

**Published:** 2022-08-16

**Authors:** Li-Yu Hong, Chii-Hong Lee, I-Jen Chiu

**Affiliations:** ^1^Division of Nephrology, Department of Internal Medicine, Shuang Ho Hospital, Taipei Medical University, New Taipei City, Taiwan; ^2^Department of Internal Medicine, School of Medicine, College of Medicine, Taipei Medical University, Taipei, Taiwan; ^3^TMU-Research Center of Urology and Kidney, Taipei Medical University, Taipei, Taiwan; ^4^Department of Anatomical Pathology, Taipei Institute of Pathology, Taipei, Taiwan

**Keywords:** minimal change disease, COVID-19 vaccination, race, disproporionality, adverse (side) effects

## Abstract

In this study, we report a case of *de novo* minimal change disease shortly after the second dose of the Moderna COVID-19 vaccine. A previously healthy 51-year-old Asian man presented with lower-limb edema and foamy urine 3 days after receiving the second dose of the vaccine. Laboratory data revealed the following: serum creatinine, 0.65 mg/dl; serum albumin, 1.3 g/dl; urine protein-to-creatinine ratio, 15.3 g. A renal biopsy was performed, and minimal change in the disease was confirmed. The patient achieved complete remission in the tenth week after starting treatment with prednisolone (1 mg/kg/day). Ethnic differences may influence the adverse effects of drugs and vaccines. However, there is very limited data to address the influence of ethnic diversity on disease prevalence, clinical presentation, and treatment outcomes in COVID-19 vaccine-associated glomerulonephritis.

## Introduction

More than eight billion doses of COVID-19 vaccines have been administered worldwide. Even though widespread vaccination has reduced the number of new cases and hospitalization, the efficacy and safety of the COVID-19 vaccine can vary predominantly in individuals with comorbidities and depends on age and ethnicity. Possible adverse effects include the development of renal complications, such as glomerulonephritis (GN).

The development of *de novo* podocytopathy or GN relapse after influenza or pneumococcus vaccination has previously been addressed (1, 2). An expanding volume of case studies has reported that immunoglobulin A (IgA) and minimal change disease (MCD) nephropathy are the first two most frequent GNs following COVID-19 vaccination (3). MCD, the most common cause of idiopathic nephrotic syndrome in children, accounts for 10–15% of cases in adults, with a decrease in incidence with age, while several studies reported that the median age of COVID-19 vaccine-associated MCD was 61 (4). A case series showed *de novo* MCD occurred after the first dose of the mRNA-based COVID-19 vaccine, especially the BNT162b2 mRNA vaccine (5). No data has been reported of *de novo* MCD following the Moderna vaccine in an Asian individual.

Herein, to the best of our knowledge, we report the first Asian case of *de novo* biopsy-proven MCD following the Moderna COVID-19 vaccine administration. Furthermore, we raise concerns regarding ethnic diversity in COVID-19 vaccine studies.

## Case description

A 51-year-old healthy Asian man with no underlying medical conditions presented to our clinic with a 2-week history of progressive limb edema increased abdominal girth, and abrupt foamy urine, which occurred on the third day after the second dose of the Moderna vaccine.

On examination, his blood pressure was 125/76 mmHg, and he had anasarca over the body. Laboratory investigations revealed the following: serum creatinine level, 0.65 mg/dl; serum albumin, 1.3 g/dl; LDL 220, mg/dl; 24-h urine protein, 15.3 g. Autoantibody or immunoglobulin levels were within normal range, and urine dipstick showed 4 + proteinuria without red blood cells (RBCs). He had no recent exposure to nephrotoxic medication, such as non-steroidal anti-inflammatory drugs. Hepatitis and HIV infection were also excluded.

**FIGURE 1:**
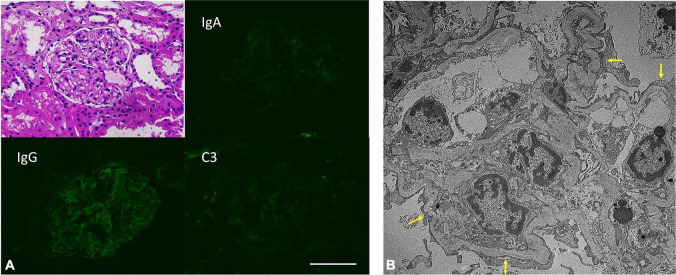
**(A)** Insignificant pathological findings in H&E staining and direct immunofluorescence staining. Scale bar: 100 μm. **(B)** Extensive podocyte foot process effacement (arrow) in patient’s kidney biopsied samples. (Electron microscopy image).

A renal biopsy was promptly performed. Pathology revealed no glomerular lesion or tubular injury and no immune deposits; however, extensive podocyte effacement and microvilli transformation confirmed an MCD diagnosis (Figure 1).

**FIGURE 2:**
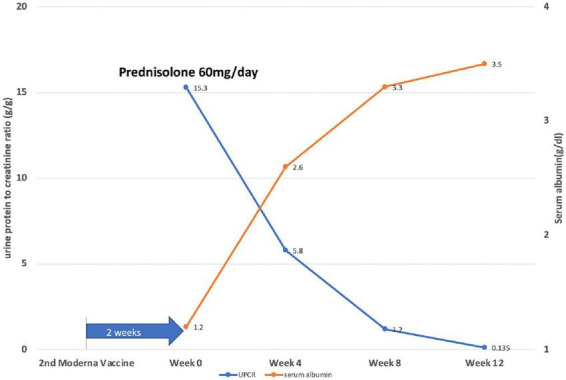
Illustration of the disease course by showing daily protein loss and serum albumin levels of the patient after 2 weeks of the second dose of the Moderna COVID-19 vaccine. Week 0: start prednisolone treatment (1 mg/kg/day).

Prednisolone was applied orally at a dose of 1 mg/kg/day, along with an angiotensin II receptor blocker. The treatment outcome was favorable. His edema resolved, and his serum albumin level rose from 1.3 to 2.6 g/dl in 4 weeks, while the serum creatinine level remained stable. The patient achieved partial remission (50% reduction from the initial value of 24-h urine protein) at 2 weeks and complete remission (urine protein excretion < 0.3g/day) at 10 weeks of steroid use (Figure 2).

We did not recommend the booster dose to the patient because of the patient’s hesitancy regarding its adverse effects. More evidence and reports are warranted to further evaluate the complexity of COVID-19 vaccine-induced MCD or another glomerulonephritis to clarify their causal relationship in the future.

## Discussion

We are the first to report regarding an Asian individual who developed *de novo* MCD after the administration of the Moderna COVID-19 vaccine. Several studies have suggested that T cell dysregulation and malfunction may be one of the driving forces of podocyte injury in MCD (6). However, the pathogenic mechanism underlying COVID-19 vaccine-associated MCD is not fully understood. It is reasonable to hypothesize that the cell immunity elicited by the COVID-19 vaccine may interact with or dysregulate T cells, promoting cytokine production, such as interleukin-2 or tumor necrosis factor α, leading to podocyte injury (7, 8). The clinical impact of evolving T-cell immunity after the COVID-19 vaccine on MCD pathogenesis remains unclear. The case series showed that the timing of MCD detection varies from one to thirteen days, with a median of 7 days after the first dose (4). Even though the onset of symptoms in our patient is 3 days right after the second dose of the COVID-19 vaccine; he may develop podocyte injury gradually after the first vaccine dose. However, neither development of *de novo* MCD nor the timing of MCD detection following the COVID-19 vaccine do not prove causation. We have several explanations to strengthen our observation. The first, the development of *de novo* podocytopathy after influenza or pneumococcus vaccination has previously been addressed as we mentioned. Second, the case series reported several *de novo* glomerulonephritis following the COVID-19 vaccine, especially IgA nephropathy and MCD (4). Moreover, we also thoroughly checked the risk factors of MCD, such as medication, infection, and major blood disorders. Finally, the onset of MCD only a few days after COVID vaccination indicates a direct link to vaccination rather than mere coincidence.

Furthermore, there is no available data suggesting which vaccine platform tends to be associated with the development of GN. Current data suggests that the mRNA-based vaccine Pfizer-BioNTech BNT162b2 has the most reported cases of MCD, followed by Moderna mRNA1273 (3, 9). Cases of MCD after receiving non-mRNA-based vaccines have also occurred (3).

The role of ethnic diversity in COVID-19 vaccine-associated adverse effects has been overlooked. Indeed, the impact of polymorphisms in host major histocompatibility complex genes on vaccine immunogenicity may raise concerns about the role of ethnic diversity in adverse effects (10). IGHV1-69 gene polymorphisms vary by ethnicity and have been shown to modulate the anti-influenza vaccine effect (11). Most COVID-19 vaccine studies did not consider ethnic diversity in protocol development or outcome studies (3). Asians accounted for only 5% of the total participants in an interim analysis of the safety and efficacy of the ChAdOx1 nCoV-19 vaccine (AZD1222) (12). Only 4.3% of the participants were Asian in a multinational study on the safety and effectiveness of the BNT162b2 mRNA COVID-19 vaccine (13). Underrepresented recruitment of ethnic minorities may lead to statistical bias or inaccuracy in the results, making them less globally applicable.

It is noteworthy that there are very few reports on the association between ethnicity and vaccine-associated adverse events. Although the MCD was reported to be more frequent in Asians, serial case studies reported COVID-19 vaccine-associated MCD mainly occurred in the Caucasian population (4). Meanwhile, the treatment course showed that most COVID-19 vaccine-associated MCD cases in the Caucasian population achieved complete remission with high-dose steroids within 1 month, while our case recovered after a more extended period. Moreover, *de novo* MCD appears to occur after the first dose of the vaccine in the Caucasian population (5, 9). We encourage further available data to address the ethnic differences in disease prevalence, clinical presentation, and treatment outcomes in COVID-19 vaccine-associated GN.

Despite more advanced research and greater clarity regarding COVID-19 and vaccination, none of the results provide sufficient evidence of safety or efficacy for the whole population. On the other hand, the rare incidence of vaccine-associated GN should not prompt COIVD-19 vaccine hesitancy. It is not only because the management strategy of COVID-19 vaccine-associated GN is consistent with conventional GN therapy, but the benefits of COVID-19 vaccination considerably outweigh the risks of glomerular disease following SARS-CoV-2 infection. Finally, the complexity of ethnicity, immunogenicity following COVID vaccination, and pathogenesis of GN development remain to be elucidated. In future studies, the importance of factoring in ethnic diversity cannot be overemphasized.

## Data availability statement

The original contributions presented in the study are included in the article/supplementary material, further inquiries can be directed to the corresponding author.

## Ethics statement

Written informed consent was obtained from the patient for the publication of pathological image or data included in this article.

## Author contributions

I-JC and L-YH conceived the research. I-JC contributed to the writing of the manuscript. I-JC and C-HL produced all the figures and visualizations. All authors contributed to the manuscript editing and approved the submitted version.
